# Clinical Analysis of Cause, Treatment and Prognosis in Acute Kidney Injury Patients

**DOI:** 10.1371/journal.pone.0085214

**Published:** 2014-02-21

**Authors:** Fan Yang, Li Zhang, Hao Wu, Hongbin Zou, Yujun Du

**Affiliations:** Department of Nephrology, The First Bethune Hospital of Jilin University, Jilin, China; University of KwaZulu-Natal, South Africa

## Abstract

Acute kidney injury (AKI) is characterized by an abrupt decline in renal function, resulting in an inability to secrete waste products and maintain electrolyte and water balance, and is associated with high risks of morbidity and mortality. This study retrospectively analyzed clinical data, treatment, and prognosis of 271 hospitalized patients (172 males and 99 females) diagnosed with AKI from December, 2008 to December, 2011. In addition, this study explored the association between the cause of AKI and prognosis, severity and treatment of AKI. The severity of AKI was classified according to the Acute Kidney Injury Network (AKIN) criteria. Renal recovery was defined as a decrease in a serum creatinine level to the normal value. Prerenal, renal, and postrenal causes accounted for 36.5% (99 patients), 46.5% (126 patients) and 17.0% (46 patients), respectively, of the incidence of AKI. Conservative, surgical, and renal replacement treatments were given to 180 (66.4%), 30 (11.1%) and 61 patients (22.5%), respectively. The overall recovery rate was 21.0%, and the mortality rate was 19.6%. Levels of Cl^−^, Na^+^ and carbon dioxide combining power decreased with increasing severity of AKI. Cause and treatment were significantly associated with AKI prognosis. Likewise, the severity of AKI was significantly associated with cause, treatment and prognosis. Multivariate logistic regression analysis found that respiratory injury and multiple organ dysfunction syndrome (MODS) were associated with AKI patient death. Cause, treatment and AKIN stage are associated with the prognosis of AKI. Respiratory injury and MODS are prognostic factors for death of AKI patients.

## Introduction

The kidney is critical in maintaining a stable internal environment by regulating the body fluid volume, maintaining electrolyte balance, and excreting potentially metabolic toxic end products. Glomerular filtration, tubular reabsorption, and tubular secretion are involved in forming urine. Glomerular infiltration involves the ultrafiltration of plasma in the glomerulus, and the filtrates, including water, salts, glucose and urea, are accumulated in the urinary space of Bowman’s capsule. Tubular reabsorption involves reabsorption of approximately 99% of the infiltrates into the blood, while tubular secretion involves the transport of substances such as K^+^, H^+^, ammonium, creatinine, and urea into the urine. The volume of urine formed is approximately 1.5 liter per day for an adult”.

Acute kidney injury (AKI), previously called acute renal failure, is characterized by an abrupt decline in renal function, resulting in an inability to secrete wastes and maintain electrolyte and water balance. [1] AKI has clinical manifestations ranging from a small elevation in serum creatinine (SCr) levels to anuric renal failure. [2] The severity of AKI is defined by Risk Injury Failure Loss End-Stage (RIFLE) and Acute Kidney Injury Network (AKIN) criteria, which are based on the presence of increased SCr levels and/or a decreased urine output. [1], [3] The AKIN definition also emphasizes the change in SCr levels over a short period of time (within 48 h). This serious disorder may aggravate pre-existing kidney disease, thus leading to a rapid loss of renal function. Several studies have shown that AKI is associated with an increased risk of morbidity and mortality.[4]–[6] The most important factor contributing to mortality after AKI is the underlying cause of AKI. [7], [8].

To reduce the severity of and improve recovery from AKI, it is important to identify the underlying cause of AKI. The etiologies of AKI are commonly categorized into prerenal, renal or postrenal. Prerenal AKI is due to impaired blood flow to the kidneys as a result of decreased blood volume, low circulating volume to the kidneys, and agents that reduce renal blood flow. Renal AKI is due to damage to the renal parenchyma, such as glomeruli, renal tubules and interstitium. Postrenal AKI is due to the obstruction of the urinary tract. The most common causes of AKI are frequently associated with infection, renal ischemia, and nephrotoxic drugs. [7], [9].

Successful management of AKI requires correction of underlying causes, management of complications, including hyperkalemia and acidosis, and timely renal replacement therapies (RRT). [9] Restoration of normal renal perfusion with intravenous fluids is the primary therapy for prerenal AKI. Relief of the obstruction is necessary to prevent irreversible kidney damage. Surgical intervention may be used to permanently remove any obstruction. In addition, fluid replacement therapy, nutritional support, avoidance of nephrotoxic agents and pharmacological intervention are used to treat AKI patients. [9], [10] RRT, such as intermittent hemodialysis (IHD), continuous renal replacement therapies (CRRT) and peritoneal dialysis (PD), have been used for AKI patients with complications such as hyperkalemia, metabolic acidosis, acute pulmonary edema and uremic symptoms. [9], [11].

Despite significant advances in supportive care, the mortality rate of AKI has not significantly improved over the past several decades. The in-hospital mortality rate is approximately 20% to 50% and may exceed 75% in critically ill patients or patients with sepsis.[12]–[14] The rate of renal recovery varies in the literature, possibly because of the lack of a consistent definition for renal recovery. Some studies reported that recovery rates ranged from 36% to 99%, based on the definition of recovery as dialysis independence at discharge. [4], [15], [16] Ali *et al.* [*17*] reported a rate of 68% for complete recovery and 5% for partial recovery, based on the definition of recovery as a decrease in SCr levels to baseline levels.

In this study, we retrospectively investigated 271 patients with AKI from December, 2008 to December, 2011. We categorized our patients according to the severity of AKI based on the AKIN criteria. The purpose of this study was to analyze clinical data, cause, treatment and prognosis of AKI patients with different AKI stages.

## Materials and Methods

### Patients

The study was approved by the ethics committee of The First Bethune Hospital of JiLin University, JiLin, China. All data used in this retrospective study were collected during the course of routine medical care in the hospital, and patients were not asked to give informed consent at the time of these clinical encounters. Since the study was retrospective and patient data were all de-identified, the Ethics Committee waived the need for written informed consent (No. 2011–12). All the data used in this study were previously collected, and use of these data did not pose any additional risks to the patients. This retrospective study included 271 patients (172 males and 99 females) admitted to our hospital and diagnosed with AKI from December, 2008 to December, 2011. We included all patients with AKI except those who had only one SCr measurement. Clinical data such as age, gender, admission and discharge times, days of hospitalization, and history of allergies, smoking, and drinking were recorded for all patients. We also evaluated other clinical information, including the department in charge of hospitalization, primary diagnosis, etiology, treatment, complications, and prognosis. Laboratory tests including SCr, blood urine nitrogen (BUN), Ca^2+^, K^+^, Na^+^, Cl^−^, carbon dioxide combining power (CO_2_CP), hemoglobin (HGB), hematocrit (HCT), serum albumin (ALB), urine blood (BLO) and urine protein (Pro) were reviewed. These biological parameters were measured using an automatic biochemistry analyzer (Hitachi 76000, Japan). The laboratory data at the first or latest abnormal SCr were used for analysis. BUN and SCr levels at peak and at discharge were also recorded. Kidney size was calculated from radiological imaging after AKI diagnosis was analyzed. The normal kidney size in Chinese patients was 10.5–11.5 cm (length)×5.0–7.2 cm (width)×2.0–3.0 cm (thickness). Enlarged kidney was defined as a length >11.5, a width >7.2 cm, or a thickness >3.0 cm. A reduced-sized kidney was defined as a length <10.5, a width <5.0 cm, or a thickness <2.0 cm.

### Definition and Criteria of AKI

AKI was defined as an absolute increase in SCr of at least 26.5 µmol/l **(**0.3 mg/dl) or a 50% or above increase in SCr from baseline according to the AKIN criteria. [3] The severity of AKI was defined by the AKIN staging criteria as follows: Stage I, SCr increase to 1.5–2 fold of baseline; Stage II, SCr increase to 2–3 fold of baseline; and Stage III, SCr increase to >3 fold of baseline or an absolute increase of >356.6 µmol/l (4.0 mg/dl) with an acute increase of at least 44.2 µmol/l (0.5 mg/dl). All patients who needed dialysis were categorized into Stage III. [3] AKI was classified as prerenal, renal or postrenal AKI according to the identified causes. The prerenal causes were categorized as follows: (a) decreased cardiac output or loss of blood due to myocardial infarction, arrhythmia, ischemic heart diseases, cardiomyopathy, hypertensive diseases or corpumonale; (b) decreased intravascular fluid volume due to diarrhea, vomiting, severe burn, or pancreatitis; (c) hypoproteinemia; (d) shock; (e) hepatorenal syndrome; and (f) use of mannitol. The renal causes were categorized as follows: (a) AKI in chronic kidney disease (A/C); (b) infection; (c) drugs; (d) multiple organ dysfunction syndrome (MODS); (e) tumor; (f) interstial nephritis; (g) nephrotoxin exposure; (h) septicemia; (i) epidemic hemorrhagic fever (EHF); (j) radiocontrast agents; (k) ketoacidosis; (l) hemolytic-uremic syndrome; (m) rhabdomyolysis; and (n) disseminated intravascular coagulation (DIC). The postrenal causes included obstructions distal to the collecting systems due to tumors, stones, urethral structure, prostate hypertrophy, or neurogenic bladder.

### Treatment of AKI

Patients were categorized according to the treatment for AKI as follows: conservative treatment; surgical treatment; and renal replacement therapy (RRT). RRT included intermittent hemodialysis (IHD), continuous renal replacement therapy (CRRT) and peritoneal dialysis (PD). Surgical treatment included surgical removal of obstructive tumors, stones, or prostate hypertrophy. Patients without RRT and surgical treatment were included in the conservative treatment group. Treatment included correction of the primary cause of AKI, fluid replacement therapy, and nutritional support. IfAKI was due to serious trauma, heart failure and acute hemorrhage, patients were administered normal saline to correct intravascular volume depletion. In AKI patients with severe hyperkalemia, 5–10 units of insulin and 50% dextrose were used to promote uptake of potassium into cells. Supportive therapies such as antibiotics and adequate nutrition were given according to standard management practice. All medications that could potentially affect renal function were discontinued.

### Outcome of AKI

Renal recovery was defined according to the SCr levels at discharge as follows: 1) complete recovery, defined as a decrease in SCr levels to the baseline value; 2) partial recovery, defined as a decrease in SCr levels compared with peak SCr, but the final Scr level was above baseline; and 3) no recovery, defined by no decrease in SCr levels compared with peak SCr. The outcomes of patients were classified as follows: (a) complete recovery; (b) partial recovery; (c) no recovery; (d) discharge without treatment, defined as discharge without comprehensive treatment in the hospital; and (e) death during hospitalization.

### Statistical Analysis

Analyses were performed using SPSS 17.0 software (SPSS, Chicago, IL, USA). Quantitative data with normal distribution were expressed as means ± SD and analyzed using analysis of variance (ANOVA). Quantitative data without normal distribution were expressed as median and interquartile range and analyzed using the rank sum test. Categorical data were expressed as frequency and percentage and analyzed using a chi square analysis. Ranked data were expressed as frequency and composition and analyzed using a Wilcoxon rank sum test. Spearman’s partial correlation tests were used to analyze the association between AKI causes and the prognosis and treatment after adjusting for age and sex. Multivariate logistic regression was used when more than one independent variable was involved. Any variable having a significant univariate test was selected as a candidate for the multivariate analysis. *P*<0.05 was considered statistically significant.

## Results

### Clinical Characteristics of AKI Patients

The baseline characteristics of the 271 AKI patients are shown in Table 1. 172 (63.5%) were male and 99 (36.5%) were female. The median age was 54 years (interquartile range, 39–68 years). The median length of hospital stay was 8 days (interquartile range, 3–16 days). Hospitalization occurred in the Department of Internal Medicine, Department of Surgery, ICU and Division of Nephrology for 142 (52.4%), 90 (33.2%), 39 (14.4%), and 40 (14.8%) patients, respectively. The number of patients with a history of allergy, smoking, or drinking alcohol was 15 (5.5%), 88 (32.5%), or 49 (18.1%), respectively.

**Table 1:** The baseline characteristics of the 271 AKI patients.

Characteristics	Patients (n = 271)
sex	
Male n (%)	172 (63.5%)
female n (%)	99 (36.5%)
length of hospital stay (interquartile range)	8 days (3–16 days)
Patients in department of hospitalization	
Department of Internal Medicine (%)	142 (52.4%)
Department of Surgery (%)	90 (33.2%),
ICU (%)	39 (14.4%)
Division of Nephrology (%)	40 (14.8%)
History	
Allergy (%)	15 (5.5%)
Smoking n (%)	88 (32.5%),
Drinking alcohol (%)	49 (18.1%)
SCr levels (µmol/l)	
First abnormal median SCr (interquartile range)	303.2 (167.4–553.5)
Median SCr levels at peak (interquartile range)	491.0 (323.8–716.0)
Median SCr levels at charge (interquartile range)	235.0 114.0–488.0)
BUN levels (mmol/l)	
First abnormal median BUN (interquartile range)	17.7 (10.9–27.22)
Median BUN levels at peak (interquartile range)	28.1 (19.06–36.83)
Median BUN levels at charge (interquartile range)	15.92 (8.0–27.0)

When the first abnormal SCr values occurred, the median BUN and SCr levels were 17.7 mmol/l (interquartile range, 10.9–27.22 mmol/l) and 303.2 µmol/l (interquartile range, 167.4–553.5 µmol/l), respectively. There were 210 (71.8%) and 222 (77.4%) patients who had hematuria and proteinuria, respectively. Median SCr levels at peak and at discharge were 491.0 µmol/l (interquartile range, 323.8–716.0 µmol/l) and 235.0 µmol/l (interquartile range, 114.0–488.0 µmol/l), respectively. Median BUN levels at peak and at discharge were28.1 mmol/l(interquartile range, 19.06–36.83 mmol/l)and15.92 mmol/l (interquartile range, 8.0–27.0 mmol/l), respectively.

Kidney size was examined in 107 (39.5%) of 271 patients after AKI diagnosis. The number of patients with normal-sized, enlarged or reduced-size kidneys or with only one kidney were 85 (79.4%), 16 (15.0%), 6 (5.6%) or 6 (5.6%), respectively.

### Analysis of Causes, Treatment and Prognosis of AKI

**Figure 1:**
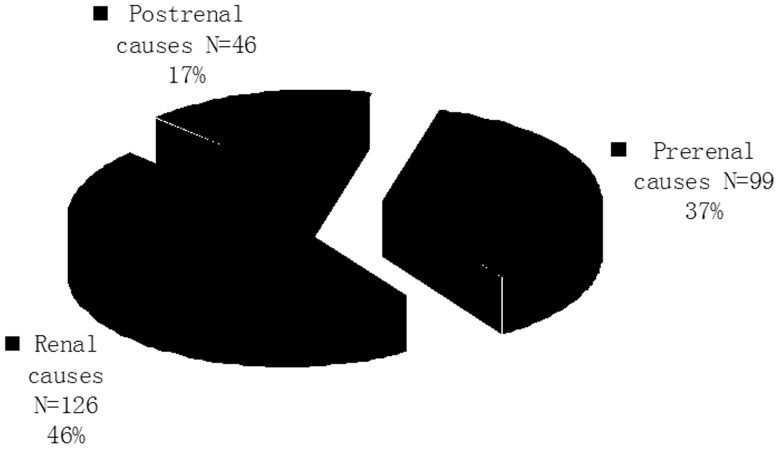
Etiologies of AKI patients. Causes of AKI were categorized into prerenal, renal, and postrental.

**Figure 2:**
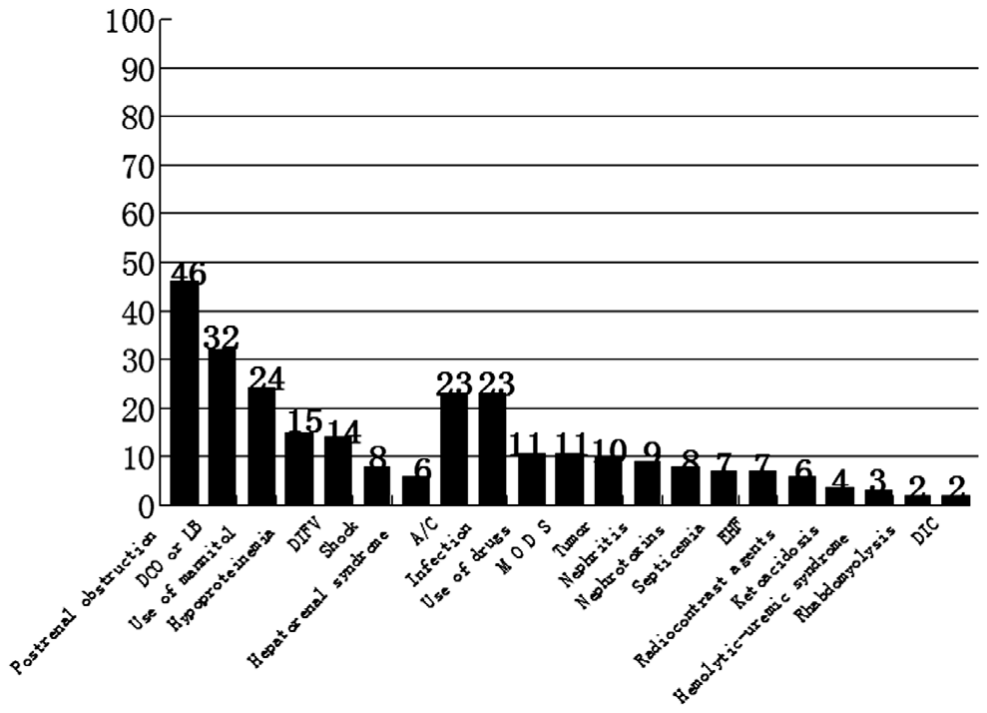
Causes of AKI in patients. From left to right:postrenal obstruction; prerenal causes, including decreased cardiac output or loss of blood (DCO or LB), use of mannitol, hypoproteinemia, decreased intravascular fluid volume (DIFV), shock, and hepatorenal syndrome; renal causes, including AKI in chronic kidney disease (A/C), infection, use of drugs, multiple organ dysfunction syndrome (MODS), tumors, nephritis, nephrotoxins, septicemia, epidemic hemorrhagic fever (EHF), radiocontrast agents, ketoacidosis, hemolytic-uremic syndrome, rhabdomyolysis, and disseminated intravascular coagulation (DIC).

The causes of AKI are summarized in Figures 1 and 2. Renal causes occurred in 126 (46.5%) patients and were the leading cause of AKI. The most frequent renal cause for AKI was chronic kidney disease (A/C) and infection (Fig. 2). Prerenal causes occurred in 99 (36.5%) patients, most commonly due to decreased cardiac output or loss of blood, which occurred in 32 patients. Mannitol also caused AKI in 24 patients. Postrenal causes were found in 46 (17.0%) patients. Of the 271 AKI patients, 30 (11.1%) underwent surgical treatment and 61 (22.5%) received RRT. The majority (66.4%) of patients received conservative treatment. After treatment, the majority of patients achieved complete recovery (57 cases; 21.0%) or partial recovery (102 cases; 37.6%). No recovery, discharge without treatment and death occurred in 22 patients (8.1%), 37 patients (13.7%) and 53 patients (19.6%), respectively.

The cause of AKI significantly correlated with the prognosis (r = −0.17, p = 0.005) and the treatment (r = 0.21, p<0.001) after adjustment for age and sex. In addition, the prognosis of AKI patients was significantly associated with the treatment (r = −0.19, P = 0.002). Postrenal causes were associated with a significantly higher recovery rate compared with prerenal and renal causes (*P*<0.0002; Table 2). The prerenal causes of AKI were associated with the highest death rate. In addition, the complete and partial recovery rates were significantly higher in patients with surgical treatment compared to those with conservative treatment and RRT treatment (*P*<0.0001; Table 2).

**Table 2:** Correlation between the cause of AKI and prognosis and treatment.

	Prognosis n (%)				
	Complete recovery	Partial Recovery	No recovery	Discharge without treatment	Death
Causes					
Prerenal (n = 99)	24 (24.2)	29 (29.3)	10 (10.1)	9 (9.1)	27 (27.3)
Renal (n = 126)	18 (14.3)	47 (37.3)	12 (9.5)	25 (19.8)	24 (19.2)
Postrenal (n = 46)	15 (32.6)	26 (56.5)	0 (0.0)	3 (6.5)	2 (4.4)
Treatment					
Conservative	41 (22.8)	39 (21.7)	19 (10.6)	33 (18.3)	48 (26.7)
Surgical	11(36.7)	19 (63.3)	0 (0.0)	0 (0.0)	0 (0.0)
RRT	5 (8.2)	44 (72.1)	3 (4.9)	4 (6.6)	5 (8.2)

RRT: renal replacement therapy. For cause: x^2^ = 32.08; *P*<0.0001 (chi square). For treatment: x^2^ = 73.57, *P*<0.0001 (chi square).

### Analysis of AKI According to AKI Stage

Table 3 summarizes the AKI stages in the cohort of 271 patients. The majority of AKI patients were Stage III (52.0%) and the minority of patients were Stage II (16.2%). No significant differences in patient age or the length of hospital stay were found among Stage I, Stage II and Stage III patients (*P*>0.05). However, the number of patients in different hospitalized departments was significantly different among patients with different AKI stages (*P*<0.01; Table 4). There were significantly more Stage III patients from the Department of Surgery, ICU and Division of Nephrology. However, more Stage I patients were hospitalized in other divisions of the Department of Internal Medicine besides Nephrology.

**Table 3:** AKI stage in 271 patients with AKI.

AKI Stage: n (%)	Median age (years)	Length of hospitalstay (days)
Stage I 86 (33.7)	59 (46–72)	10 (3–21)
Stage II 44 (16.2)	57 (38.5–68)	8.5 (2.5–15)
Stage II1 141 (52.0)	50 (37–64)	7 (4–14)
P value	0.13	0.21
H value	8.66	3.14

**Table 4:** Association* of AKI stage with department of hospitalization.

	Department of Hospitalization n(%)			
AKI stages	Internaldepartments^#^	Surgery	ICU	Nephrology
Stage I	47 (54.7)	17 (19.8)	13 (15.1)	9 (10.5)
Stage II	20 (45.5)	16 (36.4)	3 (6.8)	5 (11.4)
Stage III	35 (24.8)	57 (40.4)	23 (16.3)	26(18.4)

# Department of Internal Medicine excluding the Division of Nephrology.

*Chi square test, P = 0.003, x^2^ = 25.36.

Table 5 summarizes the association of severity of AKI with laboratory tests. No significant association of AKI stage was found with HCT, HGB, Ca^2+^, K^+^ or ALB levels (*P*>0.05). However, we found that Cl^−^, Na^+^ and CO_2_CP levels were significantly different among patients who were in different stages of AKI. There was a tendency toward a decrease in Cl^−^, Na^+^ and CO_2_CP with increased severity of AKI. The Na^+^ concentration was elevated from 337.55±28.15×10^−3^ mmol before conservative treatment to 340.48±36.20×10^−3^ mmolafter conservative treatment, from 336.30±11.73×10^−3^ mmol before surgical treatment to 347.78±13.55×10^−3^ mmol after surgical treatment, and from 335.95±22.58×10^−3^ mmol before PPT to 342.05±16.23×10^−3^ mmol after PPT. There was no significant differences in the Na^+^ concentration before and after conservative treatment (paired t test, t = 0.96, p = 0.34) and PPT (paired t test, t = 1.88, p = 0.07). Moreover, the Na^+^ concentration was significantly increased after surgical treatment (paired t test, t = 3.28, p = 0.004). In addition, SCr levels at first abnormality, at peak, and at discharge were significantly higher with increased severity of AKI. BUN levels at first abnormality and at peak, but not at discharge, were significantly higher with an increased severity of AKI (Table 6).

**Table 5:** Association of AKI stage with laboratory tests.

	Stages			
	Stage I	Stage II	Stage III	P Value
HCT	0.36 (0.30–0.41)	0.37 (0.31–0.42)	0.34 (0.27–0.40)	0.11
HGB (g/l)	122±32	124±29	118±32	0.42
Ca^2+^(×10^−3^ mmol)	5.275 (4.7–5.7)	5.225 (4.725–5.55)	5.075 (4.725–5.575)	0.33
K^+^ (×10^−3^ mmol)	10.55 (9.325–11.575)	10.6 (8.775–11.7)	10.675 (9.55–12.425)	0.21
Na^+^ (×10^−3^ mmol)	343.0 (331.0–352.5)	337.5 (325.75–352.75)	335 (320.25–344.5)	0.01
Cl^−^ (×10^−3^ mmol)	255 (240.25–269.0)	249.75 (235.5–265.25)	247.25 (232.25–257.5)	0.01
CO_2_CP (×10^−3^ mmol)	54±16.75	54.25±13.75	49.5±14.25	0.04
ALB (g/l)	28.7±8.4	27.8±9.2	28.6±6.9	0.81

These biological parameters were measured using an automatic biochemistry analyzer (Hitachi 76000, Japan).

HCT: H = 4.38 (rank sum test); Ca^2+^: H = 2.21 (rank sum test); K^+^: H = 3.13 (rank sum test); Na^+^: H = 10.49 (rank sum test); Cl^−^: H = 10.34(rank sum test); HGB: F = 0.88 (ANOVA); CO_2_CP: F = 03.25 (ANOVA); ALB: F = 0.22 (ANOVA).

**Table 6:** Association of AKI stages with BUN and SCr levels.

	Stage			
	Stage I	Stage II	Stage III	P value*
First abnormality				
BUN (mmol/l)	11.1 (8.1–15.5)	15.2 (10.9–22.5)	24.8 (16.9–33.8)	<0.0001
SCr (µmol/l)	148.1 (132.0–178.0)	260.2 (237.1–305.1)	536.3 (381.1–804.0)	<0.0001
Peak				
BUN (mmol/l)	23.9 (14.6–34.1)	24.2 (15.4–33.4)	31.0 (22.8–40.6)	<0.0001
SCr (µmol/l)	290.0 (203.6–399.9)	392.6 (267.5–543.2)	670.7 (488.7–917.8)	<0.0001
Discharge				
BUN (mmol/l)	12.5 (7.3–29.0)	18.5 (7.6–26.8)	15.8 (8.4–25.9)	0.65
SCr (µmol/l)	185.0 (101.0–323.0)	244.6 (106.0–440.1)	299.0 (135.1–540.0)	0.001

These biological parameters were measured using an automatic biochemistry analyzer (Hitachi 76000, Japan).

*P for trend among AKI stage I to III.

BUN at first abnormality: H = 71.42 (rank sum test); BUN at peak: H = 19.19 (rank sum test); BUN at discharge: H = 0.85 (rank sum test); SCr at first abnormality: H = 169.62 (rank sum test); SCr at peak: H = 114.82 (rank sum test); SCr at discharge: H = 14.41, (rank sum test).

The causes of AKI were significantly associated with the severity of AKI (*P*<0.01; Table. 7). Prerenal and renal causes were significantly associated with Stage I and II compared with postrenal causes. In addition, postrenal causes were more highly associated with Stage III compared with Stage I and Stage II patients.

Treatment of AKI was significantly associated with the severity of AKI (*P*<0.0001; Table 7). Since patients who received dialysis were categorized as Stage III, no Stage I or II patients were categorized in the RRT group. Stage I and II patients received primarily conservative treatment. The incidence of surgical treatment was significantly higher in Stage III patients compared with Stage I and II patients.

**Table 7:** Association of AKI stage with the causeof AKI, treatment, and prognosis.

	Causes n (%)				Treatment n (%)				Outcomes n (%)					
AKI stages	Prerenal	Renal	Postrenal	P value*	Conservative	Surgery	RRT	P value	Complete Recovery	Partial recovery	No Recovery	Discharge without treatment	Death	P value*
Stage I	40 (46.5)	41 (47.7)	5 (5.8)	0.0002	82 (95.4)	4 (4.65)	0 (0.00)	<0.0001	23 (26.7)	13 (15.1)	6 (7.0)	10 (11.6)	34 (39.6)	<0.0001
Stage II	21 (47.7)	19 (43.2)	4 (9.1)		41 (93.2)	3 (6.82)	0 (0.00)		13 (29.5)	10 (22.8)	7 (15.9)	7 (15.9)	7 (15.9)	
Stage III	38 (26.9)	66 (46.8)	37 (26.3)		57 (40.4)	23 (16.3)	61 (43.3)		21 (14.9)	79 (56.0)	9 (6.4)	12 (8.5)	12 (8.5)	

*p for variation among sub-groups.

RRT: renal replacement therapy. For causes: x^2^ = 22.59 (chi square). For treatment: x^2^ = 93.58 (chi square). For prognosis: x^2^ = 63.28 (chi square).

The outcome of AKI was also significantly associated with the severity of AKI (*P*<0.0001; Table 7). Rates of complete recovery and rates of death were significantly higher, and rates of partial recovery were significantly lower in Stage I patients compared with Stage III patients. In addition, univariate analysis found that respiratory injury, DIC, shock and MODS were associated with the prognosis of AKI (Table 8). Multivariate logistic regression analysis found that respiratory injury (odds ratio, 21.4) and MODS (odds ratio, 19.1) were associated with the incidence of death of AKI patients (both p<0.05).

**Table 8:** Prognostic factors for AKI.

	Prognosis n (%)				
	Complete recovery	Partial recovery	No recovery	Discharge without treatment	Death
Cardiac injury	3 (10.3)	8 (27.6)	3 (10.3)	6 (20.7)	9 (31.0)
Hepatic injury	3 (9.38)	11 (34.4)	4 (12.5)	5 (15.6)	9(28.1)
Respiratory injury*	2 (6.25)	6 (18.8)	5 (15.6)	6 (18.8)	13 (40.6)
shock△	4 (14.81)	1 (3.70)	1 (3.70)	6 (22.2)	15 (55.6)
MODS△	2 (11.8)	2 (11.8)	0 (0.00)	2 (11.8)	11 (64.7)
DIC*	0 (0.00)	0 (0.00)	1 (25.0)	0 (0.00)	3 (75.0)
Severe infection	7 (18.4)	11 (29.0)	5 (13.2)	8 (21.1)	7 (18.4)
Septicemia	1 (12.5)	1 (12.5)	0 (0.00)	2 (25.0)	4 (50.0)
Gastrointestinal bleeding	1 (14.3)	3 (42.9)	0 (0.00)	1 (14.3)	2 (28.6)
Malignant tumors	8 (26.7)	8 (26.7)	6 (20.0)	4 (13.3)	4 (13.3)
History of allergy	4 (26.7)	6 (40.0)	0 (0.00)	1 (6.67)	4 (26.7)
History of smoking	16 (18.2)	40(45.5)	4 (4.55)	11 (12.5)	17 (19.3)
History of drinking	7 (14.3)	23 (46.9)	2 (4.08)	7 (14.3)	10 (20.4)
Anemia	21 (18.0)	45 (38.5)	8 (6.84)	19 (16.2)	24 (20.5)
Renal diseases	5 (21.7)	9 (39.1)	2 (8.70)	2 (8.70)	5 (21.7)

*P<0.05;

△ *P*<0.0001 (chi square).

## Discussion

AKI is associated with a high risk of mortality and morbidity, especially in critically ill patients. AKI is common in the clinic, occurring in 8% of all in-hospital patients and in approximately 50% of ICU patients. [18] The definition of AKI according to the AKIN criteria is an absolute increase of more than 0.3 mg/dl or at least a 50% increase in SCr levels from baseline. Our study used this definition to diagnose AKI.

Of the 271 AKI patients, the majority of patients were over 50 years of age (mean age of 54 years). However, there was no association of patient age with the severity of AKI. Since AKI was defined by an abrupt increase in SCr levels within 48 h, the potential age-related and chronic effects on AKI were excluded. Of 107 patients whose kidney size was examined after AKI diagnosis, 85 (79.4%) patients had normal-sized kidneys, 16 (15.0%) patients had enlarged kidneys and 6 (5.6%) patients had reduced-size kidneys. Our finding that only 15.0% of AKI patients had an enlarged kidney suggests that AKI does not necessarily lead to an enlarged kidney. The fractional excretion of Na^+^ (FENa) is a good measure to evaluate the underlying causes of kidney enlargement in AKI patients. However, since the urinary concentrations of Na^+^ and Cr in these AKI patients were not measured in our AKI patients, we could not retrospectively calculate FENa evaluate whether the enlargement was associated with glomerular thickening. In addition, more Stage I and II patients were hospitalized in other divisions of the Departments of Internal Medicine other than the Division of Nephrology, and more Stage III patients were hospitalized at the Division of Nephrology, ICU and Department of Surgery. The cases of AKI in the Division of Nephrology were mainly associated with chronic kidney diseases, those in the Department of Surgery with obstruction, and those in the ICU with MODS or toxic agents. The cases of AKI in these departments were serious and associated with a rapid decline in kidney function, and often required dialysis. Thus, AKI patients in these departments were categorized as Stage III. In contrast, AKI in other departments was usually caused by low blood volume or the use of mannitol or radiocontrast agents. In these patients, SCr levels were transient without a rapid decline within 48 h. Thus, these patients were categorized as Stage I or II.

Prerenal, renal and postrenal causes occurred in 99 (36.5%), 126 (46.5%) and 46 (17.0%) patients, respectively. The most frequent causes for AKI in our study were postrenal obstruction (46 cases), decreased cardiac output or loss of blood (32 cases), use of mannitol (24 cases), A/C (23 cases), and infection (23 cases). The high incidence of postrenal causes of AKI in this study waspossibly due to a high incidence of kidney stones in our local area and a large number of patients with kidney stones in the Department of Surgery. Decreased cardiac output or loss of blood volume was mostly due to trauma, surgery or postpartum hemorrhage. “Osmotic nephrosis” induced by an overdose of mannitol, which is commonly used for lowering intracranial pressure, was likely the major reason for the high incidence of AKI in our study.[19]–[21] Sepsis has been reported to occur in approximately 19% of patients with moderate AKI and 23% of patients with severe AKI. [22] Consistent with a previous report, [22] we also found a relatively high incidence of infection-associated AKI. Infection has been reported to be associated with a high risk of mortality. [12].

AKI patients were categorized according to the AKIN criteria. [1], [3] Stage III patients accounted for more than half (52.0%) of our patients. The large number of Stage III patients was likely due to the inclusion of all patients with dialysis according to the AKIN criteria. [1], [3] SCr and BUN levels were also significantly elevated with increasing severity of AKI. Although SCr and BUN levels are affected by many factors, such as high protein diet, high metabolism rate, and inflammation, our results suggest that SCr and BUN levels are still good indicators for the diagnosis of AKI. In addition, Cl^−^, Na^+^, and CO_2_CP levels were also lower with increasing severity of AKI, suggesting that AKI with greater severity is likely associated with complications such as hypochloremia, hyponatremia and metabolic acidosis.

The cause of AKI was significantly associated with the severity of AKI. Prerenal and renal causes were significantly associated with Stage I and II, while postrenal causes were associated with Stage III. Since prerenal causes such as low blood volume and decreased cardiac output can induce compensatory regulation in the body, prerenal AKI can be mild, especially within a short period of time (within 48 h), and thus is categorized as Stage I according to the AKIN criteria. In contrast, postrenal obstruction produces a rapid and serious kidney injury within a short period of time without compensatory regulation, and is thus categorized as Stage III. Dialysis is often used in renal AKI caused by nephritis, nephrotoxins and rhabdomyolysis, and are categorized as Stage III. [3] Therefore, renal and postrenal causes are the major causes for Stage III AKI patients. In this study, treatment and severity of AKI were significantly associated. Stage I and II patients primarily received conservative treatment, and rarely had surgery. Surgical treatment was significantly increased in Stage III patients.

In-hospital death occurred in 53 patients (19.6%). This mortality rate is similar or lower than the mortality rate of approximately 20%–50% of the reports in the literature.[12]–[14] Prerenal causes were associated with the highest death rate. The high mortality was possibly due to serious pre-existing diseases such as shock, low blood volume after a major operation, and hepatorenal syndrome. Pre-existing comorbidities have been reported to be associated with mortality after AKI. [23] Postrenal AKI was associated with a higher recovery rate, most likely because kidney damage caused by obstruction is transient and mild and is reversible after removal of obstruction.

Using multivariate logistic regression analysis, we further found that respiratory injury and MODS were prognostic factors for death in AKI patients. Oxygen is critical for the maintenance of normal function of vital organs including the kidneys. The kidneys have a very rich blood supply and are very sensitive to ischemia and hypoxia. Therefore, the kidneys are susceptible to damage in patients with respiratory injury, and thus AKI patients with respiratory injury show a poor prognosis. In addition, long-term respiratory failure can lead to multiple organ failures due to insufficient oxygen supply. MODS can also be caused by severe trauma, septic shock, and accidents. With the increase in the number of failed organs, the mortality rate increases. [24] It has been reported that mortality rate is significantly increased in the presence of MODS. [25], [26] In agreement with these reports, we also found that MODS was a prognostic factor for death in AKI patients.

In summary, we retrospectively studied 217 hospitalized patients with a diagnosis of AKI. Prerenal causes were the leading cause for AKI in this study. The majority of patients received conservative treatment, and only 22.5% of patients received RRT. The recovery rate was 21.0% and the mortality rate was 19.6%. Both cause and treatment were significantly associated with the prognosis of AKI. Similarly, the severity of AKI was significantly associated with cause, treatment and prognosis. Respiratory injury and MODS are prognostic factors for death in AKI patients.
